# *Muscari comosum* L. Bulb Extracts Modulate Oxidative Stress and Redox Signaling in HepG2 Cells

**DOI:** 10.3390/molecules26020416

**Published:** 2021-01-14

**Authors:** Fabiana Giglio, Maria Antonietta Castiglione Morelli, Ilenia Matera, Chiara Sinisgalli, Rocco Rossano, Angela Ostuni

**Affiliations:** Department of Sciences, University of Basilicata, 85100 Potenza, Italy; giglio.fabiana01@gmail.com (F.G.); maria.castiglione@unibas.it (M.A.C.M.); ilenia.matera@gmail.com (I.M.); chiara.sinisgalli@gmail.com (C.S.)

**Keywords:** *Muscari comosum* L., aqueous and methanol extracts, phenolic compounds, flavonoids, HepG2 cells, ROS, redox pathways, ABC transporters, NMR metabolomics analysis

## Abstract

*Muscari comosum* L. bulbs are commonly used as food in South Italy and also in folk medicine. By evaluating in vitro antioxidant activity and biological activities of their aqueous and methanol extracts, we shed light on the potential role, including both the nutraceutical and health benefits, of this plant. Total polyphenol content (TPC) and total flavonoid content (TFC) were evaluated by the Folin–Ciocalteu method and by the aluminum chloride method, respectively. Antioxidant activity was investigated by three in vitro assays and relative antioxidant capacity index (RACI) was calculated to compare results obtained by different tests. The extracts were tested to evaluate their possible involvement in redox homeostasis, using the human hepatoma (HepG2) cell line used as model. The extracts exhibited concentration/solvent dependent radical scavenging activity, as well as dysregulation of some genes involved in redox pathways by promoting Nrf2, SOD-2, GPX1, ABCC6 and ABCG2 expression. NMR metabolomics analysis suggests that HepG2 cells treated with *Muscari comosum* extracts experience changes in some metabolites involved in various metabolic pathways.

## 1. Introduction

The increase in life expectancy has led to an increase, not only in Europe but also worldwide, in the proportion and in the absolute number of elderly people, which in turn has led to a rise in the prevalence of diseases linked to aging (cancer, neurodegenerative diseases and metabolic disorders). Starting from the last decade of the last century, many efforts have been made to identify the properties and potential applications of nutraceutical substances [1,2]. The area of greatest interest in eating habits as a prototype of a useful diet for the prevention and treatment of diseases related to aging is the Mediterranean area. The Mediterranean diet is characterized by high consumption of fruits and vegetables, moderate consumption of fish and reduced consumption of cheeses and meats. Wild or semi-cultivated plants play a key role in this diet [3]. Although the Mediterranean diet has been the subject of numerous studies, the contribution provided by wild or semi-cultivated plants has often been neglected. They are widely consumed in the daily diet of the Mediterranean people; their consumption is often associated with information on a potential therapeutic effect based on the knowledge of popular medicine. Only recently has there been an increasing interest in these plant species; in particular several studies have shown their phytonutrient content and potential therapeutic effect [3,4].

*Leopoldia comosa* L. (syn *Muscari comosum* L.) is a spontaneous plant belonging to the Asparagaceae family that grows in the whole Mediterranean area, including the Basilicata region (Italy). The use of *Muscari comosum* bulbs has a long tradition in Greece, the Middle East and the Eastern Mediterranean [5,6]. The tradition of its specific use as a food has been studied in recent ethnobotanic surveys in Sardinia, southern Italy and central Turkey [7]. In folk medicine, *Muscari comosum* bulbs were used for the treatment of toothache, to remove stains, or as an anti-inflammatory, diuretic and aphrodisiac [8]. They have a high variety of nutrients: starch (8%), nitrates (1000 ppm) and simple sugars including sucrose (0.5%), fructose (0.3%), glucose (0.07%) and arabinose (0.04%). Its peculiarity is represented by the abundance of minerals, such as potassium, phosphorus, calcium, iron, copper, manganese and magnesium. Vitamin C is only present in trace amounts [9].

Since the 1980s, Adinolfi et al. carried out intensive phytochemical research on *Muscari comosum* extracts, identifying homoisoflavanones (above all 3-benzyl-4-cromanones) [10], terpenes and glycosylated triterpenes [11] as the main metabolites. In addition to their great phytochemical importance, homoisoflavanones have a wide range of biological activities: in vitro, some exhibit anti-inflammatory, analgesic, and hypocholesterolemic properties, as well as antiallergic, antihistamine and antimutagenic properties.

Homo-isoflavones have antioxidant activity by scavenging free radicals, blocking lipid peroxidation and inhibiting the enzyme xanthine-oxidase [12,13]; moreover, these polyphenols exhibit an anti-bacterial and antifungal activity as they are able both to inhibit the growth of *Aspergillus niger* and *Penicillium chrysogenum* and to block the enzymes involved in proliferation of *Phytosporaparasitica* during infection [14]. Recent studies show that the homoisoflavanones contained in this plant, interacting with the estrogenic receptors, may have a potential role as hormone substitutes or useful supplements for the treatment of hormone-sensitive tumours [15]. Recently, it has also been shown that the extract of *Muscari comosum* has potential antioxidant and hypoglycaemic activity due to its inhibitory activity on α-amylase and α-glucosidase [16].

Until now, few studies have investigated their biological activity in a cell model. In a study conducted by Casacchia et al., *M. comosum* bulbs showed antioxidant activity, an inhibitory effect on α-amylase and pancreatic lipase, and antiproliferative activity on mammalian adenocarcinoma cells, showing that it is a potential complement to the treatment of chronic diseases and cancer [17].

In this study, we investigated the in vitro antioxidant potential of three different extracts of *Muscari comosum* and evaluated their biological activity on HepG2 hepatoma cells in order to clarify the cellular mechanisms underlying the observed effects. Furthermore, we evaluated their effects on the redox balance and on the expression levels of some genes involved in the cell’s redox system. The metabolomics analysis performed on HepG2 cell lysates and culture media reveals that *M. comosum* extracts modify the intra- and extra-cellular metabolic profile.

## 2. Results

### 2.1. Total Polyphenol and Flavonoid Content of M. Comosum Extracts (MCE)

*M. comosum* bulbs were extracted with different solvents obtaining different extracts: methanol:water (70:30 *v*/*v*) (MET70), methanol:water (50:50 *v*/*v*) (MET50), and pure water (WT). The extraction yield ranged from 7.83% to 10.22%; the highest value was achieved with water as solvent, whereas, MET70 extract showed the lowest value (Table 1). Total phenolic content (TPC) of three extracts, evaluated by the Folin–Ciocalteu method, was expressed as mg of gallic acid equivalents (GAE)/g of extract. Among the samples analyzed, the MET70 extract had the highest content (58.72 ± 1.11 mg GAE/g), followed by the other two extracts that showed the same phenolic content (52.66 ± 1.80 and 54.01 ± 0.83, for WT and MET50, respectively).

Total flavonoid contents (TFC), determined by the aluminum chloride method, were reported as mg of quercetin equivalents/g of extract. The aqueous/methanolic extracts (19.70 ± 0.96 mg QE/g for MET50 and 20.37 ± 1.24 mg QE/g for MET70, respectively) showed higher content than the WT extract (14.99 ± 0.70 mg QE/g). TFC represented about 33% of the total polyphenols.

### 2.2. Antioxidant Activity In Vitro

In this study, the antioxidative potential of different extracts obtained from MEC was evaluated by various antioxidant assays, including total reducing power, 2,2-diphenyl-1-picrylhydrazyl (DPPH), nitric oxide and superoxide radicals scavenging activities.

Total reducing power is one of the methods commonly used for the analysis of antioxidant capacity of plant extracts; it measures spectrophotometrically the reduction of Fe^3+^ to Fe^2+^. Results (Table 1) ranged from 37.93 ± 1.55 to 47.52 ± 2.86 mg of gallic acid equivalents (GAE)/g of extract. As well as for flavonoid and phenolic acid content, the aqueous/methanolic extracts showed higher values than aqueous extract.

**Figure 1:**
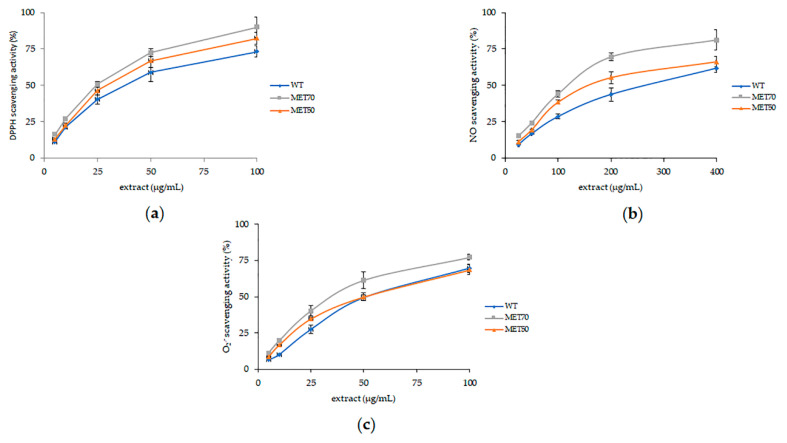
Radical scavenging activity. Determination of DPPH (**a**), ˙NO (**b**) and O_2_^−^ (**c**) radical scavenging activity of *M. comosum*. All experiments were performed in triplicate. Data are expressed as mean ± SD (*n* = 6), *p* < 0.05) for all tested dosages.

Radical scavenging activity was evaluated vs. DPPH, a synthetic radical, and vs. biological radicals, NO and O_2_^−^. Results were expressed as IC_50_, were IC_50_ values indicate the sample concentration (μg/mL) required to scavenge 50% of free radicals (Table 1). All bulb extracts were able to scavenge DPPH radicals in a dose-dependent manner (Figure 1). MET70 extract was the most active at neutralizing radicals, reporting the lowest IC_50_ value (24.60 ± 2.13 μg/mL), followed by MET50 and WT extracts.

Nitric oxide radical (˙NO) is a ubiquitous free radical involved in different physiological functions; however, when it is abundant, it is also implicated in inflammation and other pathological conditions. As shown in Figure 1, all samples were shown to possess nitric oxide scavenging activity in a concentration-dependent manner. MET70 extract exhibited the best antioxidant activity with an IC_50_ of 122.94 ± 7.06 μg/mL.

The radical scavenging activity of the extracts was confirmed against O_2_^−^ radicals, known as a major cause of cellular oxidative stress. MET70 reported the lowest IC_50_ value with no statistical differences with ascorbic acid used as a reference (Table 1).

**Figure 2:**
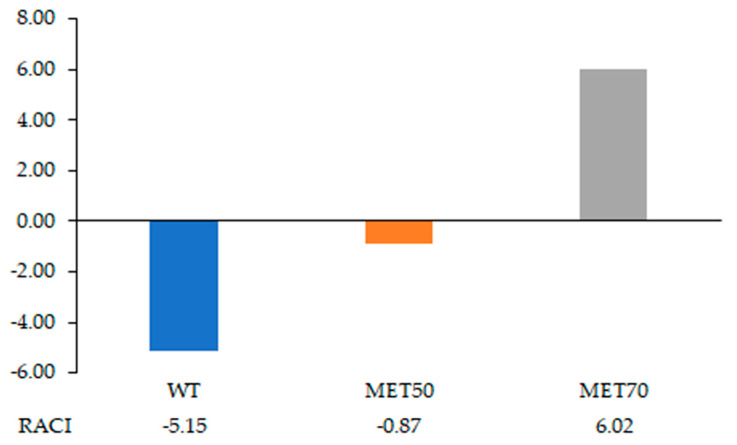
Relative antioxidant capacity index (RACI) of methanol: water (70:30 *v*/*v*) (MET70), methanol: water (50:50 *v*/*v*) (MET50), pure water (WT) extracts.

To compare data obtained by different chemical methods used to evaluate the antioxidant activity, the relative antioxidant capacity index (RACI) has been proposed [18]. RACI is created from the perspective of statistics by integrating the antioxidant capacity values generated from different in vitro methods. The RACI of each bulb extract was calculated by averaging the standard scores transformed from raw data generated with different antioxidant methods (TPC, TRC, DPPH, NO and O_2_^−^). The standard score represents the distance between the raw data and the mean in units of the standard deviation, which is negative when the raw data are smaller than the mean and positive when larger. Results of antioxidant activity expressed as IC_50_ were transformed to 1/IC_50_, before the RACI determination. Hydro-alcoholic solvents were allowed to extract higher amounts of polyphenols and flavonoids, responsible for the antioxidant activity of extracts [19,20]. MET70 showed the highest levels of TPC and TFC and consequently, the highest index: 6.02, followed by MET50: −0.87 and WT: −5.15 (Figure 2).

### 2.3. Effect of M. Comosum Extracts on Cell Viability

**Figure 3:**
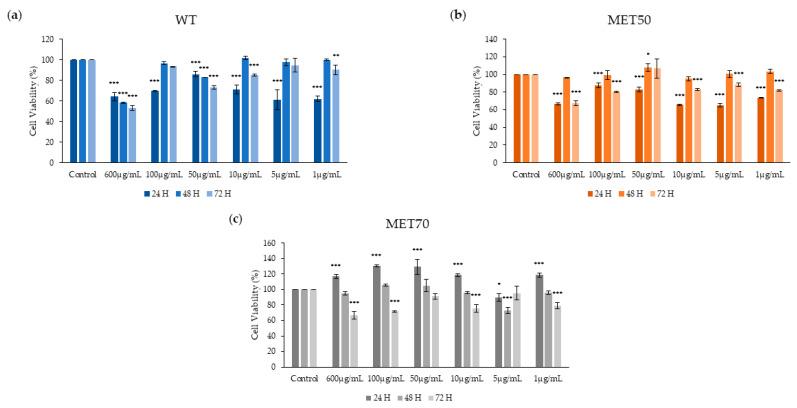
*M. comosum* extracts effect on HepG2 cells viability. Cells were treated with different concentrations of the extracts: (**a**). pure water (WT); (**b**). MET50; (**c**). MET70 for 24, 48, 72 h. Viability of each sample was expressed as the percentage with respect to control cells (0.2% DMSO, vehicle). Data are expressed as the mean ± SD of three independent experiments (*n* = 3). Comparisons between treatments and control groups were performed by one-way ANOVA followed by Dunnett *post-hoc* correction. * *p* < 0.033; ** *p* < 0.002; *** *p* < 0.001.

Cell viability of MCE was evaluated by MTT assay on the HepG2 cell line used as the model. Whatever extract was administered to the cells, only a reduction in viability of around 20% was observed. WT extract reduced cell viability at 600 μg/mL after 24, 48 and 72 h (Figure 3a), as well as the highest dose of MET50 after 24 and 72 h (Figure 3b). MET70 increased cell proliferation at the highest doses (600–100–50 μg/mL) after 24 h (Figure 3c). The opposite effect occurred after 72 h, MET70 reduced cell viability (Figure 3c).

**Figure 4:**
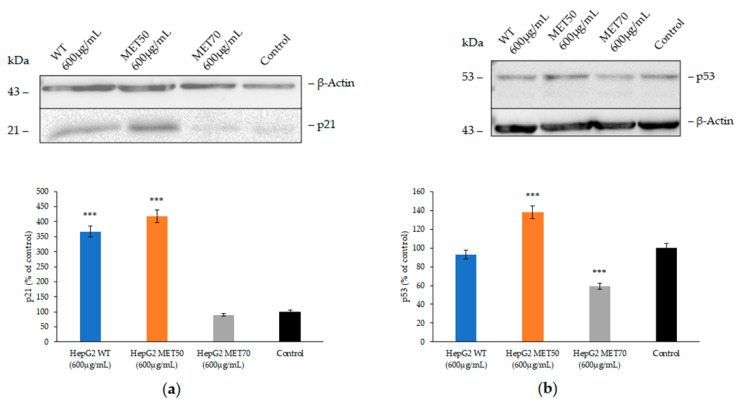
Effect of *M. comosum* extracts on p21 and p53 expression of HepG2 cells. Representative western blot of p21 (**a**) and p53 (**b**) in cells treated with 600 μg/mL extract or DMSO (control) for 24 h. Densitometric analysis of the immunoreactive bands are expressed as the mean ± SD of three independent experiments (*n* = 3). The protein levels were normalized with β-actin content. Data were normalized to control cells set to 100%. Comparisons between treatments and control groups were performed by one-way ANOVA followed by Dunnett *post-hoc* correction. *** *p* < 0.001.

No change in morphology or necrosis was observed in cells treated with the three extracts up to 72 h. WT and MET50 at 600 ug/mL for 24 h increased the expression of the cyclin-dependent kinase inhibitor p21^cip1/waf1^, which is known to trigger cell growth arrest [21] (Figure 4a). Interestingly, the increase of p21 expression is p53-independent or -dependent in cells treated with WT or MET50, respectively (Figure 4b). No change in p21 and a decrease of p53 expression were observed in MET70-treated cells.

### 2.4. Effect of M. comosum Extracts on Intracellular Reactive Oxygen Species

**Figure 5:**
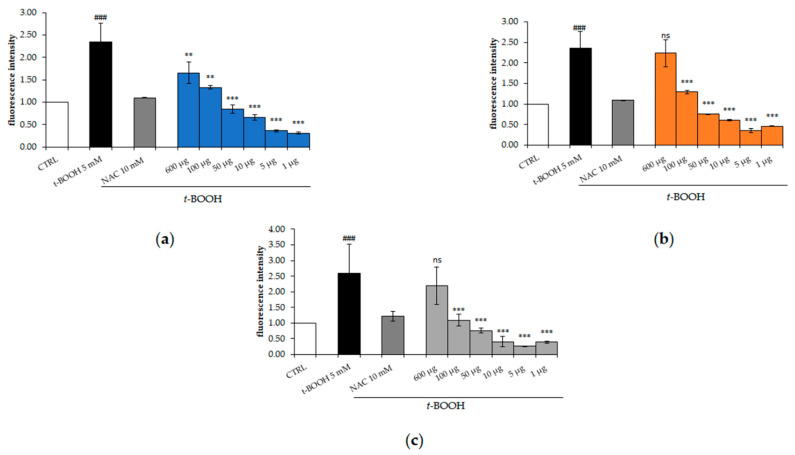
Effects of MEC on *t*-BOOH-induced intracellular reactive oxygen species (ROS) generation. Cells were pre-treated with (**a**) WT, (**b**) MET50 and (**c**) MET70 extracts at different concentrations for 24 h and subsequently incubated with 5 mM *t*-BOOH for 1 h. ROS generation was measured by DCFH-DA staining with flow cytometry analysis. Data are expressed as the mean ± SD of three independent experiments (*n* = 3). Student’s *t*-test was used for statistical analysis. ### *p* < 0.001 versus CTRL, *** *p* < 0.001, ** *p* < 0.01 versus *t*-BOOH-treated cells, ns: not significant.

The antioxidant activity of *M. comosum* extracts was evaluated in the HepG2 cell line. In particular, cells were treated with different doses (600, 100, 50, 10, 5 and 1 μg/mL) of each MCE for 24 h and then oxidative stress was induced by *t*-BOOH, known as a source of ROS. HepG2 cells stressed with *t*-BOOH showed a higher amount of ROS showing an increase in fluorescence, as compared to untreated cells (Figure 5). All of the extracts showed the best antioxidant activity at low doses, comparable to NAC or even more than NAC, a known antioxidant. As shown in Figure 5b,c, cells treated with 600 µg/mL of MET50 and MET70 reported fluorescence values not statistically different from stressed cells; on the contrary, the WT extract partially reduces the intracellular ROS at a concentration of only 600 µg/mL (Figure 5a). Pre-treatment with low doses (100–1 μg/mL) of the extracts for 24 h protected cells from oxidative stress.

### 2.5. Effect of M.Comosum Bulb Extracts on Several Markers Involved in Oxidative Stress

**Figure 6:**
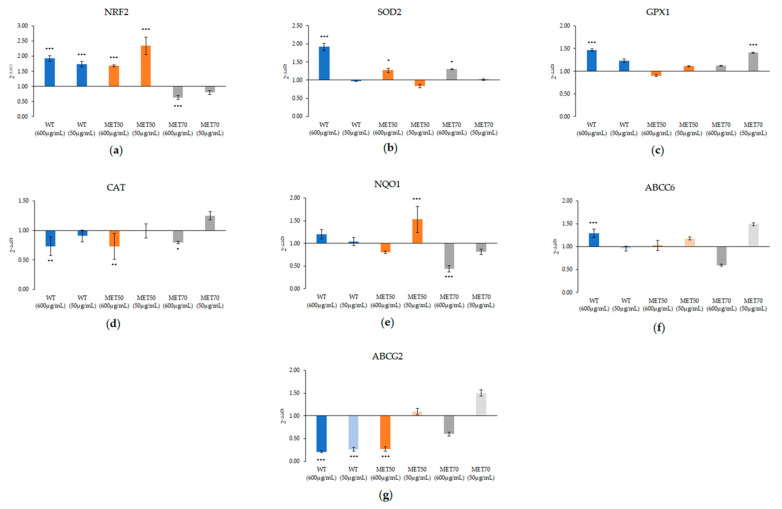
Effect of MEC on (**a**) Nuclear factor-erythroid 2 p45-related factor 2 (NFR2), (**b**) Superoxide dismutase (SOD2), (**c**) Glutathione peroxidase (GPX1), (**d**) Catalase (CAT), (**e**) NADPH quinone oxidase-1 (NQO1), (**f**) ATP binding cassette subfamily C member 6 (ABCC6), (**g**) ATP binding cassette subfamily G member 2 (ABCG2) expression. Gene expression was normalized to β-actin mRNA levels. Data are expressed as fold changes, normalized with respect to the related untreated control. Results are expressed as the mean and 95% confidence interval of three different experiments. Any statistical analysis was performed on ΔCt values by using one-way ANOVA followed by Dunnett *post-hoc* correction. * *p* < 0.033; ** *p* < 0.02; *** *p* < 0.001.

The expression of different genes was evaluated in order to investigate molecular mechanism involved in biological activity of MEC (Figure 6).

After 24 h of treatment, WT promoted the expression of nuclear factor-erythroid 2 p45-related factor 2 (NRF2), superoxide dismutase (SOD-2) and glutathione peroxidase (GPX1), especially at the highest dose (600 ug/mL). Otherwise, MET50 and MET70 acted at the lowest dose (50 µg/mL). MET50 increased the expression of NRF2 and NQO1, MET70 improved mainly GPX1 expression as well as ABCC6 and ABCG2 expression. No extract affected catalase expression (Figure 6).

### 2.6. Effect of M. Comosum Extracts on Metabolite Composition of Cells and Corresponding Culture Media

We examined HepG2 cell lysates alone or after treatment with the three *M.comosum* extracts at 50 μg/mL concentration in order to trace any metabolic variation in intracellular metabolites induced by the extracts. We also examined the corresponding culture media where cells were grown to characterize their composition. The metabolic profiles of cell lysates and culture media were analyzed by NMR spectroscopy and Chenomx Profiler was used to identify the metabolites presents in cells and their culture media.

**Figure 7:**
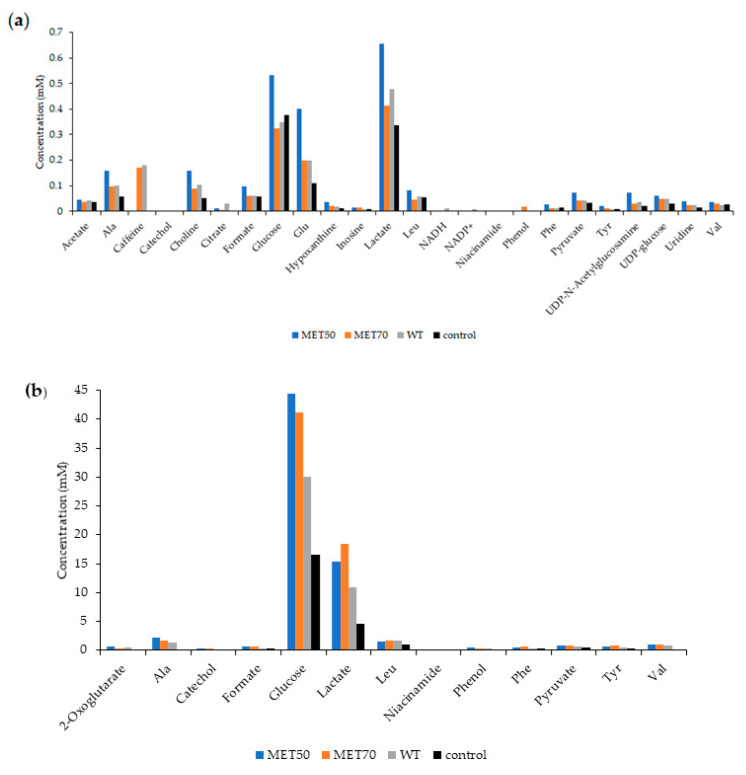
Metabolites identified in HepG2 cells (**a**) and their culture media (**b**). Concentration data (mM) were obtained by Chenomx Profiler.

In cell lysates, 24 metabolites were identified and quantified from their resonances in ^1^H NMR spectra: six amino acids (Ala, Leu, Glu, Phe, Tyr, Val); five carboxylic acids (acetate, citrate, formate, lactate, pyruvate); three carbohydrates or key intermediates in their metabolism (glucose, UDP-glucose, UDP-Nacetylglucosamine); three nucleic acid bases or nucleosides (hypoxanthine, inosine, uridine); two coenzymes (NADH, NADP^+^); two alcohols (catechol, phenol); caffeine, and choline. Niacinamide (Vitamin B3) was present only in MET50 extract, though at very low concentration (0.001 mM) (Figure 7a). The treatment with MET50 extracts results in higher amounts of almost every metabolite.

In culture media treated with *M. comosum* extracts, we identified 13 metabolites: five amino acids (Ala, Leu, Phe, Tyr, Val); four carboxylic acids (2-oxo-glutarate, formate, lactate, pyruvate); one carbohydrate (glucose); two alcohols (catechol, phenol); and vitamin B3 (Figure 7b). Also in this case, the treatment with MET50 extracts results in higher quantity of many metabolites, while the MET70 extracts produce higher contents of formate, lactate, pyruvate, phenylalanine, tyrosine and niacinamide.

## 3. Discussion

The aim of this study was to characterize on a cell model the biological activities of *Muscari comosum*, a spontaneous plant whose bulbs are used in the Mediterranean diet and in folk medicine.

We investigated the antioxidant activity of *Muscari comosum* L. bulbs extracts obtained by maceration with three different solvent mixtures. The increase of solvent polarity leads to a greater extraction yield, probably due to the major solubility of carbohydrates and proteins, as reported by several authors [22,23]. Moreover, an increase in solvent polarity and aqueous mixture improved the extraction of polyphenols [18,24], compounds that are known to show potential health benefits [25]. MET50 and MET70 showed higher amounts of polyphenols and flavonoids compared to WT extract.

Total phenolic and flavonoid contents found in this study are more similar with those reported by Loizzo et al. (2010) [13] and with those reported in our previous work for the aqueous methanolic bulb extract [16]. As reported by several authors [19,20], the amount of phenolic compounds represents an important indicator of antioxidant capacity. Among these, flavonoids are the most abundant polyphenols in plants, they show antioxidant activity and radical scavenging capacity, providing beneficial health effects [18]. MET70 had the highest amounts of total polyphenols and flavonoids and demonstrated the best radical scavenging activity vs. DPPH and O_2_^−^. In a previous study [16], twelve different compounds were identified in the HPLC profile of 70% methanol extract of *M. comosum* bulbs, seven of them were phenolic acids (gallic, vanillic, chlorogenic, caffeic, syringic, *p*-coumaric acid and ferulic) and five were flavonoids (catechin, epicatechin, rutin, naringenin and kaempferol).

The HepG2 cell line was used as model to study the biological activities of these extracts. Cells treated with the same concentration of MET50 and WT showed a similar trend in viability as a function of time. A significant decrease of viability after 24 h, followed by an increase after 48 h incubation and again a significant decrease after 72 h of incubation was observed. On the other hand, MET70 increased cell proliferation after only 24 h, but a further decrease of viability at longer times was observed. No change in morphology or necrosis was observed. Western blot analysis of p53 and p21 of cells treated for 24 h with the highest concentration of the extract suggested an increase in p21 expression p53-dependent or not for WT and MET50, respectively. However, in MET70-treated cells, no change in p21 expression and a decrease of p53 were observed, which could explain the increased cell proliferation after 24 h. ROS are one of the most relevant mediators in cell cycle arrest and may be responsible for the cycle arrest in WT- and MET50-treated cells. However, other mechanisms must be invoked to explain why a high concentration of MET70 leads to an increase in cell viability at 24 h, while causing an increase of ROS [26]. Further investigations are in progress.

The extract protected cells from oxidative stress by reducing intracellular ROS especially at lowest doses (100–1 μg/mL). MET50 and MET70, increased ROS levels at 600 µg/mL without a statistical difference from *t*-BOOH-treated cells, probably due to the higher content of phenolic compounds. Polyphenols can act as antioxidants or pro-oxidants, depending on the concentration, pH and the cellular environment. Many studies reported that caffeic acid, ferulic acid, catechin, epigallocatechins and other phenolic acids and flavonoids can induce ROS production directly or by acting on other pathways [27].

Different activity at different doses was also confirmed by qRT-PCR. It is established that many transcription factors, including nuclear factor erythroid 2-related factor 2 (Nrf2), are activated by ROS and regulate the redox status of cells [28]. Because it regulates a wide spectrum of antioxidant and detoxification genes, Nrf2 provides a principal inducible defense against oxidative stress [29] by modulating levels of intracellular antioxidant enzymes. WT, the extract with lowest polyphenol content, promoted Nrf2 expression and, consequently, the upregulation of SOD2 and GPX1 but only at the highest dose. Otherwise, MET50 and MET70 showed antioxidant activity at lowest doses by acting on different markers. In particular, MET50 induced a considerable increase of Nrf2, which, in turn, resulted in upregulation of downstream target genes SOD-2, GPX-1 and NQO1. MET70 upregulated Nrf2 and GPX-1 but mainly ABCC6 and ABCG2 expression. ABCC6 and ABCG2 are two ATP dependent transporters involved in chemotherapy and multidrug resistance [30,31,32,33] but they are also responsible for glutathione transport, which is important to maintain redox state in cells [34,35,36].

Several ATP binding cassette (ABC) transporters are Nrf2 targets. They contribute to xenobiotic defense mechanisms and are important determinants of xenobiotic uptake, distribution, and excretion [37]. In HepG2 cells treated with the MET70 extract, which showed the best antioxidant activity both in vitro and in cells, the observed increase of mRNA of ABCG2 suggested its contribution to decrease the oxidative stress. Interestingly, ABCC6 also showed the same trend, confirming its involvement in oxidative stress regulation.

The metabolomics analysis performed on HepG2 cell lysates and culture media reveals that *M. comosum* extracts modify the intra- and extra-cellular metabolic profile. The involved metabolic pathways concern different biomolecules, such as carbohydrates, carboxylic acids, pirimidines, amino acids, and coenzymes. Further studies will be required to elucidate the effects of *M. comosum* on this complex network of metabolic interactions.

## 4. Materials and Methods

### 4.1. Chemicals

Absolute methanol, sodium phosphate monobasic (NaH2PO4), Dulbecco’s modified Eagle medium (DMEM), dimethyl sulfoxide (DMSO), [3-(4,5-dimethyl-2-thiazolyl)-2,5-diphenyl-2H-tetrazolium bromide] (MTT), 20,70-dichlorodihydrofluorescein diacetate (DCFH-DA), *N*-acety-*L*-cystein (NAC), tert-butyl hydroperoxide (*t*-BOOH), deuterated water, and 3-trimethylsilyl propionic acid-d_4_ sodium salt (TSP) were purchased from Sigma Aldrich S.p.A. (Milan, Italy). Trypsin-EDTA solution, fetal bovine serum (FBS), glutamine, penicillin-streptomycin, and phosphate saline buffer (PBS) were purchased from Euroclone (Milan, Italy). Reagents used for RT-PCR were purchased from Euroclone (Milan, Italy). Acetonitrile and formic acid were purchased from Merck (Merck KGaF, Darmstadt, Germany).

### 4.2. Bulb Extracts

*Muscari comosum* L. were harvested in Basilicata (Southern Italy). Bulbs were peeled, cleaned with distilled water, frozen at −20 °C, then lyophilized and finally grinded into a fine powder, placed in three different extraction solvents: pure water (WT), methanol:water (70:30 *v*/*v*) (MET70), and methanol:water (50:50 *v*/*v*) (MET50) in a 1:7 (*w*/*v*) ratio, and incubated in orbital stirring at 20 °C for 24 h. The soluble fraction was recovered by centrifugation (18,000× *g* for 10 min at 20 °C), while the insoluble fraction was macerated again in the extracting solutions (ratio 1:5 *w*/*v*). After centrifugation, the two extracts were pooled, frozen and lyophilized.

### 4.3. Determination of Total Phenol and Flavonoid Contents

Total phenolic (TPC) content of bulb extracts was determined spectrophotometrically with the Folin–Ciocalteu method. To 0.1 mL of extract, were added 0.5 mL of the Folin–Ciocalteu reagent (diluted 10 times with water) and, after 3 min, 0.4 mL of 7.5% sodium carbonate was added. After 2 h of incubation at room temperature in the dark, the absorbance was measured at 765 nm using an Ultrospec 2000 spectrophotomer (Pharmacia Biotech, Uppsala, Sweden). TPC was determined as gallic acid equivalents (GAE) and expressed in terms of mg GAE/g of extract.

For the determination of total flavonoid content (TFC), 0.15 mL of bulb extracts were added to 0.45 mL methanol, 0.03 mL 10% aluminium chloride, 0.03 mL 1 M potassium acetate and 0.85 mL of distilled water and incubated for 30 min at room temperature. Then the absorbance was read at 415 nm [38], using quercetin as a standard. TFC was determined as quercetin equivalents (QE) and expressed in terms of mg QE/g of extract.

### 4.4. Free Radical Scavenging Activity

The capacity of the bulb extracts to scavenge 2,2-diphenyl-1-picrylhydraziyl (DPPH˙) radical was evaluated as previously reported [16]. Briefly, 0.5 mL of the extracts were added to 0.5 mL of 0.2 mM DPPH˙ in methanol, and incubated at room temperature for 30 min in the dark. The absorbance was measured at 517 nm. Antioxidant activity was expressed as IC_50_, where IC_50_ values indicate the sample concentration (μg/mL) required to scavenge 50% of DPPH free radicals. Gallic acid was used as the standard. DPPH radical scavenging activity (%) = [(Abs_control_ − Abs_extract_)/Abs_control_] × 100.

The nitric oxide (˙NO) radical scavenging activities was determined by adding 100 μL of extracts to 100 μL of 20 mM sodium nitroprusside and incubating it for 60 min at room temperature. Then, 100 μL of Griess reagent was added to the mixture and successively, the absorbance was measured at 560 nm after 10 min of incubation in the dark.

The superoxide radical (O_2_^−^) scavenging activity was determined by adding 100 μL of extracts to 100 μL of 2 mM NADH and 100 μL 50 µM nitrotetrazolium blue. Then, 100 μL of 50 µM phenazine methosulphate was added to the mixture, and after 5 min of incubation at room temperature the absorbance was read at 560 nm.

Radical scavenging activities (%) = [(Abs_control_ − Abs_extract_)/Abs_control_] × 100. For both, results were expressed as IC_50_. Ascorbic acid was used as a positive control [16].

### 4.5. Total Reducing Power

For the total reducing power (TRP) assay, 0.1 mL of different concentrations of bulb extracts were mixed with 0.5 mL of 0.2 M sodium phosphate buffer pH 6.6 and 0.5 mL of 1% potassium ferricyanide. After 30 min of incubation at 50 °C, 0.5 mL of 10% trichloroacetic acid was added to the mixture and then centrifuged (10 min at 4000× *g*). Afterwards, 0.5 mL of the supernatant was mixed with 0.5 mL of distilled water and 0.1 mL of 0.1% ferric chloride. The absorbance was read at 700 nm. TRP was expressed in terms of mg GAE/g of extract [16].

### 4.6. Cell Culture and Treatments

Human hepatoblastoma cells (HepG2) were maintained in Dulbecco’s modified Eagle’s medium (DMEM) containing 25 mM glucose, supplemented with 10% fetal bovine serum (FBS), 2 mM L-glutamine, 100 U/mL penicillin and 100 µg/mL streptomycin at 37 °C, in an atmosphere humidified with 5% of CO_2_. The methanol and the aqueous extracts were dissolved at 2 mg/mL in dimethyl sulfoxide (DMSO) 0.2% (*v*/*v*) and sterile water, respectively. Control cells were treated at the same final percentage of DMSO. The stock solutions were diluted with DMEM to the desired concentrations immediately before use.

### 4.7. Cell Viability Assay

Cell viability was determined using the standard colorimetric assay for mitochondrial reductase catalyzed reduction of yellow MTT (3-(4,5-dimethylthiazol-2-yl)-2,5-diphenyltetrazolium bromide) to give a purple formazan product. HepG2 were seeded at a density of 1.5 × 10^4^ in a 96-well culture plate treated with increasing concentrations of the extracts ranging from 10 µg/mL to 600 µg/mL for 24, 48 and 72 h. MTT reduction was quantified by measuring the light absorbance at 570 nm, with background subtraction at 630 nm, using a microplate reader (Multiskan^TM^ Go Microplate Spectrophotometer, Thermo Scientific, Waltham, MA, USA). The value of OD_570_-OD_630_ is proportional to the number of viable cells in each well. The percentage viability of treated cells was calculated with the help of the following formula, % viability of cells = (average optical density of treated cells/average optical density of control cells) × 100%, while the cell viability in the control group was considered 100%. Each test was repeated three times in triplicate.

### 4.8. Western Blot Analysis

HepG2 cells were suspended in RIPA buffer (50 mM Tris–HCl pH 8, 150 mM NaCl, 1% Igepal, 0.2% SDS, 1% sodium deoxycholate) supplemented with a protease and phosphatase inhibitors cocktail and lysed by sonication. Then, lysates were centrifuged at 13,000 rpm for 10 min at 4 °C. The supernatant, resuspended in Laemmli sample buffer (60 mM Tris–HCl pH 6.8, 10% glycerol, 2% SDS, 1% β-mercaptoethanol and 0.002% bromophenol blue), was resolved on 12% SDS-PAGE gels and proteins were transferred to nitrocellulose membrane. Membranes were blocked for 1 h with saturation buffer (5% nonfat dried milk in PBS or TBS with 0.05% Tween 20) and then probed with primary antibody overnight at 4 °C, 1:400 anti-β-Actin (Sigma-Aldrich, St. Louis, MO, USA), 1:500 anti-p21 (Santa Cruz, Dallas, TX, USA), 1:100 anti-p53 (Biolegend, San Diego, CA, USA). Membranes were washed three times with PBST or TBST and incubated with appropriate horseradish peroxidase-conjugated secondary antibody at room temperature for 1 h and signal visualized by ECL™ Western Blotting Detection Reagents (GE Healthcare, Chicago, IL, USA) or SuperSignal™ West Pico PLUS Chemiluminescent Substrate (Thermo Scientific), using Chemidoc TM XRS detection system equipped with Image Lab Software for image acquisition (Bio-Rad, Hercules, CA, USA). Densitometric analysis was performed by using GelAnalyzer 2010 software. Protein expression level in the control sample was taken as 100%. Each result was expressed as percentage of the value of the control sample. Each test was repeated three times.

### 4.9. Measurement of the Intracellular Reactive Oxygen Species

The intracellular ROS level was measured with 2′,7′-dichlorodihydrofluorescein diacetate (DCFH-DA) [22]. HepG2 cells were plated at a density of 1 × 10^4^ cells/well in 24-well plates, incubated with different concentrations of *M. comosum* L. extracts or *N*-acetyl-*L*-cystein (NAC 10 mM) for 24 h, and stressed with 5 mM of *tert*-butyl hydroperoxide (*t*-BOOH) for 1 h. Finally, the cells were stained with 10 µM DCFH-DA for 30 min at 37 °C in the dark, and fluorescence was measured by BD FACSCanto II (BD Pharmingen, San Jose, CA, USA) at an excitation wavelength of 485 nm and emission wavelength of 515–540 nm.

### 4.10. Real-Time Reverse Transcription PCR (qRT-PCR)

HepG2 cells were cultured in the presence of the three extracts or 0.2% DMSO (vehicle) for 24 h; then cells were harvested and total RNA was extracted using Quick-RNA MiniPrep kit (Zymo Research, Irvine, CA, USA), according to the manufacturer’s protocol. cDNA was synthesized using a High-Capacity cDNA Reverse Transcription Kit (Applied Biosystem, Foster City, CA, USA) in accordance with the manufacturer’s instructions. Real-time quantitative RT-PCR was performed with a 7500 Fast Real-Time PCR System (Applied Biosystems) using iTaq^TM^ Universal-SYBR^®^ Green Supermix (Bio-Rad). To confirm PCR specificity, the PCR products were subjected to a melting-curve analysis. The comparative threshold cycle method (∆∆Ct) was used to quantify relative amounts of product transcripts with β-actin as endogenous reference control. Primers were designed for spanning exon–exon junctions, eliminating undesirable genomic DNA amplification (Table 2).

### 4.11. Sample Preparation for NMR Analysis

The HepG2 cell line was cultured in 6-well plates at a density of 8.5 × 10^5^/well and treated with 50 μg/mL of MCE. After 24 h, culture medium and cells (≈5 million of cells per treatment) were collected and preserved at −80 °C until use. For the metabolite extraction, cells were suspended in phosphate buffer saline (500 uL, PBS, pH 7.4) w/o Ca^2+^ and Mg^2+^ and kept on ice for 30 min. Then, cells were lysed through sonication (37%, 30 s) and centrifuged (10 min, 4 °C, 13,000 rpm) to remove precipitated cellular debris. The supernatant was transferred to a fresh extraction tube, followed by the addition of methanol, chloroform and distilled water in the ratio of 4:4:2.85. The aqueous phases were transferred to a new tube and lyophilized for NMR analysis [39].

Lyophilized samples were mixed with 600 μL of D_2_O and 5 μL of TSP (3-trimethylsilyl propionic acid-d_4_ sodium salt) and placed in a 5 mm NMR tube. TSP was used as both chemical shift reference and internal standard for quantitative analysis. All samples were at pH 7. The NMR spectra were acquired on a Varian Unity Inova 500 MHz spectrometer. The spectrometer was equipped with a 5-mm triple resonance pulsed field *z*-axis gradient probe, operating at 499.96 MHz for ^1^H. The temperature during all experiments was kept at 25 °C. No sample rotation was applied.

The 1D ^1^H NMR spectra were acquired using a solvent suppression pulse sequence to saturate the residual ^1^H water proton signal. One hundred and twenty-eight transients were acquired with a spectral width of 5995 Hz and an acquisition time of 4 s. A recycle delay of 1 s was selected. VNMRJ 2.1B software (Agilent Technologies, Santa Clara, CA, USA) was used to acquire all the spectra. Spectra were processed using NMR SUITE 8.0 (Chenomx Inc., Edmonton, AB, Canada). 1D spectra were Fourier transformed with a FT size of 32 k and a 1 Hz line-broadening, phased and a polynomial baseline correction was applied over the whole spectral range. The PROFILER module was used to identify metabolites by fitting the compound signatures from the spectral Chenomx NMR Suite library. Metabolite concentrations were calculated by determining the heights of the signatures best fitting the experimental signals.

### 4.12. Statistical Analysis

At least three independent experiments were performed for statistical analysis and expressed as mean ± standard deviation (SD) of the replicate measurements. One-way ANOVA followed by Dunnett post-hoc correction was used to evaluate the statistical significance of the results and to compare the mean values from untreated control and MEC treated cells.

## 5. Conclusions

We demonstrated that different solvent extractions influenced the biological activity of *Muscari comosum* L. extracts. Methanol improved extraction yield, the amount of total flavonoids and phenolics and, consequently the antioxidant activity in vitro. This was also confirmed in a HepG2 cell line used as a model. It is interesting to underline the different effect with different concentrations of extracts. The highest polyphenol content resulted in good radical scavenging activity of MET50 and MET70, which reduced intracellular ROS in a dose-dependent manner by promoting Nrf2, SOD-2, GPX1, ABCC6 and ABCG2 expression. However, the extracts acted as pro-oxidant at higher concentrations altering different signaling pathways that could be important in a chemotherapy strategy even if further investigations are needed.

The antioxidant activities of *M. comosum* may not be attributed to a single mechanism. In fact, the NMR metabolomics analysis suggests that HepG2 cells experience some changes in their metabolism, due to the treatment with *M. comosum* extracts, as demonstrated by the increase in cell lysates of some metabolites involved in various metabolic pathways.

## Figures and Tables

**Table 1:** Extraction yield, phenols, flavonoids content and antioxidant activity of *M. comosum* extracts.

^a^ Extracts	^b^ Yield (%)	^c^ Phenols (mgGAE/g)	^d^ Flavonoids (mg QE/g)	^e^ DPPH (IC_50_ µg/mL)	^f^ ˙NO (IC_50_ µg/mL)	^g^ O_2_^−^ (IC_50_ µg/mL)	^h^ Reducing Power (mg GAE/g)
WT	^a^ 10.22 ± 0.21	^a^ 52.66 ± 1.80	^a^ 14.99 ± 0.70	^a^ 38.02 ± 1.91	^a^ 269.21 ± 11.28	^a^ 51.43 ± 2.97	^a^ 37.93 ± 1.55
MET50	^b^ 8.56 ± 0.11	^a^ 54.01 ± 0.83	^b^ 19.70 ± 0.96	^b^ 29.43 ± 2.70	^b^ 168.52 ± 15.29	^a^ 50.10 ± 5.29	^b^ 44.51 ± 2.63
MET70	^c^ 7.83 ± 0.19	^b^ 58.72 ± 1.11	^b^ 20.37 ± 1.24	^c^ 24.60 ± 2.13	^c^ 122.94 ± 7.06	^b^ 36.50 ± 3.84	^b^ 47.52 ± 2.86
Gallic acid				5.05 ± 1.85			
Ascorbic acid					39.66 ± 2.17	45.62 ± 3.01	

^a^ MET: methanol/water; WT: water. ^b^ Expressed as % yield (g of extract/g of sample). ^c,h^ Expressed as mg of gallic acid equivalent/g of extract. ^d^ Expressed as mg of quercetin equivalent/g of extract. ^e,f,g^ IC_50_: concentration (μg/mL) of sample required to scavenge 50% of radicals. Values are reported as mean ± s.e.m. of two independent experiments performed in triplicate (*n* = 6). The mean values with different letters (in the same column) are significantly different (*p* < 0.05, Tukey’s test) as analyzed ANOVA.

**Table 2:** List of primers used in this study.

Gene	Accession Number	Forward Primer	Reverse Primer
ABCC6	NM_001171.5	5′-ATCACTGATCCTTCCATCTTG-3′	5′-ACCAGCGACACAGAGAAGAGG-3′
ABCG2	NM_004827.2	5′-ATCACTGATCCTTCCATCTTG-3′	5′-GCTTAGACATCCTTTTCAGG-3′
β-actin	NM_001101.3	5′-CCTGGCACCCAGCACAAT-3′	5′-GCCGATCCACACGGAGTACT-3′
Catalase	NM_001752.4	5′-ATACCTGTGAACTGTCCCTACCG-3′	5′-GTTGAATCTCCGCACTTCTCCAG-3′
GPX1	NM_000581.4	5‘-CAGTCGGTGTATGCCTTCTCG-3′	5′-CTCGTTCATCTGGGTGTAGTCC-3′
SOD2	NM_000636.4	5′-CCGACCTGCCCTACGACTAC-3′	5′- AACGCCTCCTGGTACTTCTCC-3′
NQO1	NM_000903	5′-GGTGGTGGAGTCGGACCTCTA-3′	5′-AGGGTCCTTCAGTTTACCTGTGAT-3′
NRF2	NM_00114541.3	5′-AACTACTCCCAGGTTGCCCA-3′	5′-CATTGTCATCTACAAACGGGAA-3′

## Data Availability

Not applicable.
